# Real-world comparison of pembrolizumab and nivolumab in advanced hepatocellular carcinoma

**DOI:** 10.1186/s12885-023-11298-z

**Published:** 2023-08-29

**Authors:** Yen-Hao Chen, Ching-Hua Tsai, Yen-Yang Chen, Chih-Chi Wang, Jing-Houng Wang, Chao-Hung Hung, Yuan-Hung Kuo

**Affiliations:** 1 Division of Hematology-Oncology, Department of Internal Medicine, Kaohsiung Chang Gung Memorial Hospital and Chang Gung University College of Medicine, No.123, Dapi Rd., Niaosong Dist, 833 Kaohsiung, Taiwan; 2 School of Medicine, College of Medicine, Chang Gung University, 333 Taoyuan, Taiwan; 3 School of Medicine, Chung Shan Medical University, 402 Taichung, Taiwan; 4 Department of nursing, School of nursing, Fooyin University, 831 Kaohsiung, Taiwan; 5 Division of Trauma Surgery, Department of Surgery, Kaohsiung Chang Gung Memorial Hospital and Chang Gung University College of Medicine, 833 Kaohsiung, Taiwan; 6 Division of General Surgery, Department of Surgery, Kaohsiung Chang Gung Memorial Hospital and Chang Gung University College of Medicine, 833 Kaohsiung, Taiwan; 7 Division of Hepatogastroenterology, Department of Internal Medicine, Kaohsiung Chang Gung Memorial Hospital and Chang Gung University College of Medicine, 833 Kaohsiung, Taiwan

**Keywords:** Hepatocellular carcinoma, Pembrolizumab, Nivolumab, Immunotherapy

## Abstract

**Background:**

Nivolumab and pembrolizumab have not been directly compared in clinical trials, and the aim of this study is to investigate the efficacy and safety of nivolumab versus pembrolizumab in patients with advanced hepatocellular carcinoma (HCC) in real-world practice.

**Methods:**

We retrospectively reviewed patients with HCC who received intravenous nivolumab or pembrolizumab alone as second-line and later therapy. The objective response was determined according to the Response Evaluation Criteria in Solid Tumors criteria version 1.1. Adverse events (AEs) were graded based on the National Cancer Institute Common Terminology Criteria for Adverse Events, version 5.0. The Kaplan–Meier method was used to analyze progression-free survival (PFS) and overall survival (OS). Prognostic values were estimated using hazard ratios with 95% confidence intervals (CIs).

**Results:**

In total, 120 patients were enrolled, including 95 who received nivolumab and 25 who received pembrolizumab. All patients were staged as Barcelona Clinic Liver Cancer stage C, and 29 patients were classified as Child-Pugh classification B (7). The response rate of the pembrolizumab and nivolumab groups were 8.0% and 7.4%, respectively. There was no significant difference in the median PFS between the pembrolizumab and nivolumab groups (2.7 months versus 2.9 months). The median OS in the nivolumab group was longer than that in the pembrolizumab group (10.8 months versus 8.1 months); however, the difference was not statistically significant. The effects of pembrolizumab and nivolumab on the median PFS and OS were consistent across the subgroups based on baseline characteristics. The severity of all AEs was grades 1–2 without treatment interruption or dose adjustment; there was no statistically significant difference in the incidence of treatment-related AEs between these two groups. Additionally, the percentage of patients receiving subsequent therapy was consistent between the two groups.

**Conclusion:**

The efficacy and safety of pembrolizumab and nivolumab were comparable in the management of patients with pretreated HCC in real-world practice.

## Introduction

Hepatocellular carcinoma (HCC) is the most common type of primary liver cancer, and its incidence has increased in recent decades. In Taiwan, HCC is the fourth most common cancer and the second leading cause of cancer-related mortality [1]. Systemic treatment options for advanced HCC have rapidly evolved in recent years with the approval of several new multikinase inhibitors (MKIs) and immune checkpoint inhibitors (ICIs). Sorafenib was the first systemic therapy approved for the treatment of advanced HCC. It targets several signaling pathways involved in tumor cell proliferation, angiogenesis, and apoptosis [2, 3]. Subsequently, lenvatinib was approved as the first-line treatment for advanced HCC based on the results of the REFLECT trial [4]. In this trial, lenvatinib was not inferior to sorafenib in terms of overall survival (OS). However, lenvatinib significantly improved the objective response rate (ORR) and progression-free survival (PFS) compared with sorafenib. Recently, immunotherapy has emerged as a promising treatment option for advanced HCC. ICIs are monoclonal antibodies that target molecules on immune cells or cancer cells that regulate the immune response. The IMbrave150 trial, a randomized, open-label, phase III study, demonstrated that the combination of atezolizumab and bevacizumab significantly improved OS, ORR, and PFS compared with sorafenib and was generally well tolerated with no unexpected safety signals [5]. The IMbrave150 trial has been a game-changer in the treatment of advanced HCC, and this combination has become the first choice for first-line systemic therapy of HCC.

After the progression to sorafenib, targeted therapies and immunotherapies have emerged as promising second-line treatment options for HCC. Regorafenib and cabozantinib are both MKIs that target angiogenesis and tumor growth and have been approved as second-line treatments for patients with advanced HCC who had progressed on sorafenib based on the RESORCE and CELESTIAL trials [6, 7]. According to the results of the REACH-2 trial, ramucirumab has also been approved as a second-line treatment for HCC in patients who have progressed to or are intolerant to sorafenib with alpha-fetoprotein **(**AFP) ≥ 400 ng/mL [8]. Nivolumab and pembrolizumab are both ICIs and have been approved as second-line treatment options for patients with HCC who have progressed to or are intolerant to sorafenib. The CheckMate-040 trial, a phase I/II open-label multi-center study, demonstrated that nivolumab had an ORR of 14%, with a median duration of response of 19.4 months. The disease control rate (DCR) was 55% and the median OS was 15.1 months. The safety profile of nivolumab is consistent with that observed in other tumor types [9, 10]. In contrast, pembrolizumab was approved based on the results of the KEYNOTE-224 trial, which showed an ORR of 16% with a median response duration of 16 months. The DCR was 57%, median PFS was 4 months, and median OS was 17 months [11, 12]. Recently, a phase 3 KEYNOTE-394 study revealed that pembrolizumab alone significantly improved ORR, PFS, and OS compared to placebo as a second-line therapy for Asian patients with advanced HCC [13]. Overall, nivolumab and pembrolizumab have shown promising results as second-line treatment options for patients with HCC who have progressed to or are intolerant to sorafenib treatment.

It is important to note that nivolumab and pembrolizumab have not been directly compared in clinical trials, and there is currently no clear consensus on which drug is superior. Therefore, this study aimed to investigate the real-world efficacy and safety of nivolumab and pembrolizumab in patients with advanced HCC.

## Materials and methods

### Study design and participants

**Fig. 1:**
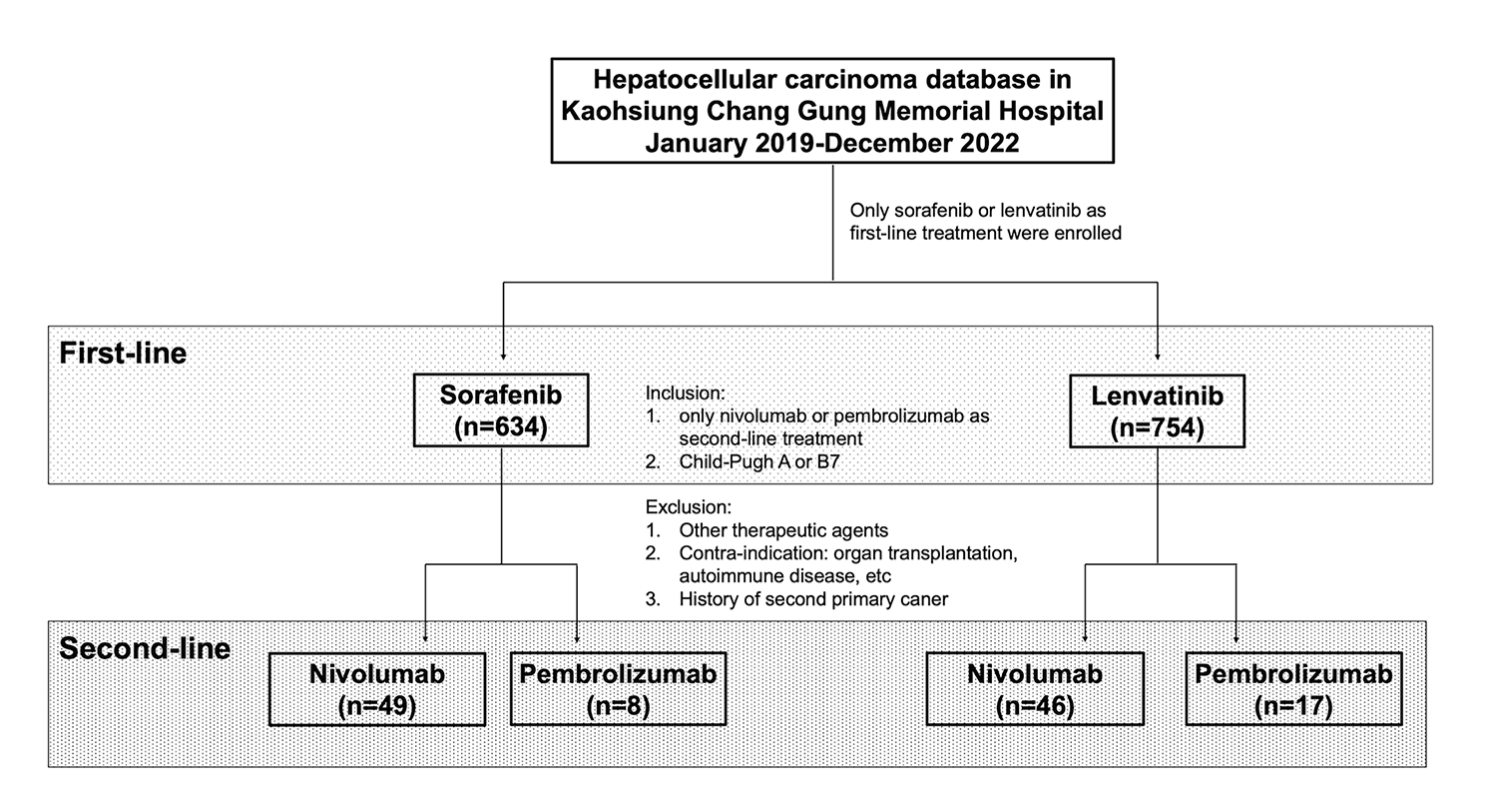
Flowchart of the process of identifying patients with advanced hepatocellular carcinoma who received nivolumab or pembrolizumab after progression on sorafenib or lenvatinib

Patients with advanced HCC who received systemic therapy between January 2019 and December 2022 at Kaohsiung Chang Gung Memorial Hospital were retrospectively reviewed. The eligibility criteria were as follows: (1) sorafenib or lenvatinib as the first-line systemic therapy with disease progression; (2) nivolumab alone or pembrolizumab alone (combination with other targeted therapies, ICIs, chemotherapy, or locoregional therapy were not allowed); (3) no history of other ICI treatment before nivolumab or pembrolizumab (atezolizumab, ipilimumab, durvalumab, and tremelimumab); (4) no history of a second primary malignancy (even hepatocholangiocarcinoma was not allowed); (5) well-known contraindications were excluded, including solid organ/peripheral stem cell transplantation, long-term use of steroid, autoimmune disease and human immunodeficiency virus infection; and (6) well-documented medical records to collect clinical information. Finally, 120 patients with advanced HCC who received nivolumab alone or pembrolizumab alone as second-line or later therapy were identified. A flowchart of identifying these HCC patients according to the inclusion and exclusion criteria is shown in Fig. 1.

### Tumor staging and assessments

HCC was diagnosed based on the pathology or non-invasive criteria of the American Association for the Study of Liver Disease (AASLD) [14, 15]. Each patient was staged according to the Barcelona Clinic Liver Cancer (BCLC) staging classification before nivolumab or pembrolizumab initiation [16]. The albumin-bilirubin (ALBI) score was calculated according to the serum albumin and total bilirubin levels using the following formula: ALBI score = (log_10_ bilirubin [mol/L]×0.66) + (albumin [g/L]×–0.085). The ALBI score was graded as: grade 1 ( ≤ − 2.60), grade 2 (− 2.59 to − 1.39), or grade 3 ( > − 1.39) [17].

Each patient was required to have at least one measurable target lesion for the assessment of treatment response using magnetic resonance imaging (MRI) or dynamic multiphase computed tomography (CT) of the liver, which was performed every 8–12 weeks after the start of nivolumab or pembrolizumab. The treatment response was independently determined by two radiologists without any medical information, in accordance with the Response Evaluation Criteria in Solid Tumors (RECIST) criteria version 1.1 [18].

### Treatment and safety

The patients received intravenous nivolumab at a dose of 3 mg/kg every 2 weeks (Q2W), intravenous pembrolizumab at a dose of 2 mg/kg, or a fixed dose of 200 mg every 3 weeks (Q3W). The use of nivolumab or pembrolizumab was determined by patients’ selection and their economic status. The treatment was continued until disease progression or intolerable adverse events (AEs). The patients were monitored at the outpatient clinic for Q2W or Q3W according to the choice of nivolumab or pembrolizumab, including AEs, serum biochemistry, thyroid function, cortisol, and blood sugar levels. The AE and immune-related adverse event (irAE) grades were evaluated based on the National Cancer Institute Common Terminology Criteria for Adverse Events (CTCAE) version 5.0 [19].

### Statistical analysis

Differences in the clinical characteristics between the two groups were determined using the chi-square test for categorical variables. PFS was defined as the time from the initiation of nivolumab or pembrolizumab treatment to the date of disease progression or death due to any cause. OS was calculated from the start of nivolumab or pembrolizumab treatment until death due to any cause or the date of the last visit. Comparison of PFS and OS between patients treated with nivolumab or pembrolizumab was performed using the Kaplan–Meier method and the log-rank test. Prognostic values were estimated using hazard ratios (HRs) with 95% confidence intervals (CIs). All analyses were conducted using the SPSS 26 software (IBM, Armonk, NY, USA). Statistical significance was set at p < 0.05. significant.

### Ethics statement

This study was approved by the Institutional Review Board of the Chang Gung Medical Foundation (202101199B0) and conducted in accordance with the Declaration of Helsinki. The requirement for written informed consent was waived by the Institutional Review Board of the Chang Gung Medical Foundation (202101199B0) because of the retrospective design of this study.

## Results

### Patient characteristics

Our study enrolled 120 patients with advanced HCC who received immunotherapy after progression with sorafenib or lenvatinib at Kaohsiung Chang Gung Memorial Hospital between January 2019 and December 2022, including 95 patients receiving nivolumab and 25 patients receiving pembrolizumab. There were 94 men and 26 women with a median age of 59 years (range: 32–84 years). All patients were classified as BCLC stage C and an Eastern Cooperative Oncology Group Performance Status score of 0 or 1. Ninety-one patients (75.8%) were classified as Child–Pugh class A, and the remaining 29 patients (24.2%) were classified as Child-Pugh class B (7). All patients had ALBI grade 1 (35.8%) or 2 (64.2%). In the analysis of viral hepatitis, 80 (66.7%) and 33 (27.5%) patients had hepatitis B and hepatitis C viral infections, respectively. Macrovascular invasion (inferior vena cava, hepatic vein, and portal vein) was found in 64 patients (53.3%), including 20 (16.7%) with portal vein thrombosis. The incidence of extrahepatic spread and lymph node (LN) metastasis was 55.0% and 34.2%, respectively. Fifty-one patients (42.5%) underwent a hepatectomy before nivolumab or pembrolizumab treatment. Pembrolizumab or nivolumab was prescribed as second-line therapy in 66 patients (55.0%) and as third-line and later therapy in 54 patients (45.0%). There were no statistically significant differences in the baseline characteristics between the nivolumab and pembrolizumab groups, including the AFP level (the median AFP level was 1378 ng/mL in the pembrolizumab group and 834 ng/mL in the nivolumab group). A comparison of the background information between the two groups is shown in Table 1.

**Table 1:** Comparison of baseline characteristics between 120 patients with advanced hepatocellular carcinoma who received pembrolizumab or nivolumab

Characteristics	Pembrolizumab (n = 25)	Nivolumab (n = 95)	P value
Age (median, range)	58 (32–83) years	61 (38–84) years	0.42
Sex			
Male	19 (76.0%)	75 (78.9%)	0.75
Female	6 (24.0%)	20 (21.1%)	
Child–Pugh classification			
A	18 (72.0%)	73 (76.8%)	0.62
B (7)	7 (28.0%)	22 (23.2%)	
BCLC classification			
C	25 (100%)	95 (100%)	
ALBI grade			
1	7 (28.0%)	36 (37.9%)	0.43
2	18 (72.0%)	59 (62.1%)	
Hepatitis B			
Yes	19 (76.0%)	61 (64.2%)	0.27
No	6 (24.0%)	34 (35.8%)	
Hepatitis C			
Yes	7 (28.0%)	26 (27.4%)	0.95
No	18 (72.0%)	69 (72.6%)	
Macrovascular invasion (IVC, HV, PV)			
Yes	14 (56.0%)	50 (52.6%)	0.76
No	11 (44.0%)	45 (47.4%)	
Main portal vein thrombosis			
Yes	4 (16.0%)	16 (16.8%)	0.92
No	21 (84.0%)	79 (83.2%)	
History of hepatectomy			
Yes	11 (44.0%)	40 (42.1%)	0.87
No	14 (56.0%)	55 (57.9%)	
Extrahepatic spread			
Yes	18 (72.0%)	48 (50.5%)	0.06
No	7 (28.0%)	47 (49.5%)	
Lymph node metastasis			
Yes	7 (28.0%)	34 (35.8%)	0.47
No	18 (72.0%)	61 (64.2%)	
Treatment lines			
2	11 (44.0%)	55 (57.9%)	0.21
≥ 3	14 (56.0%)	40 (42.1%)	
AFP (median, range) ng/ml	1378 (2.6 – > 80,000)	834 (2.1 – > 80,000)	0.10

BCLC: Barcelona-Clinic Liver Cancer; ALBI: Albumin-Bilirubin; IVC: inferior vena cava; HV: hepatic vein; PV: portal vein; AFP: alpha-fetoprotein. All status mentioned above were determined at the time of pembrolizumab or nivolumab initiation.

### Treatment response

The treatment response to nivolumab or pembrolizumab was determined based on the RECIST criteria version 1.1. In the pembrolizumab group, the ORR was 8.0%, and all patients showed partial response (PR), while 9 (36.0%) patients had stable disease (SD) and 14 (56.0%) patients had progressive disease (PD), indicating a DCR of 44.0%. In contrast, the percentages of PR, SD, and PD in the nivolumab group were 7.4%, 31.6%, and 61.0%, respectively, with a DCR of 39.0%. There were no statistical differences in the ORR, SD, and DCR between patients treated with pembrolizumab or nivolumab (P = 0.90). A comparison of treatment responses to pembrolizumab and nivolumab is presented in Table 2.

**Table 2:** Treatment response to pembrolizumab or nivolumab

	Pembrolizumab (n = 25)	Nivolumab (n = 95)	P value
Partial response	2 (8.0%)	7 (7.4%)	0.90
Stable disease	9 (36.0%)	30 (31.6%)	
Progressive disease	14 (56.0%)	58 (61.0%)	
Disease control rate	11 (44.0%)	37 (39.0%)	

### Survival

**Fig. 2:**
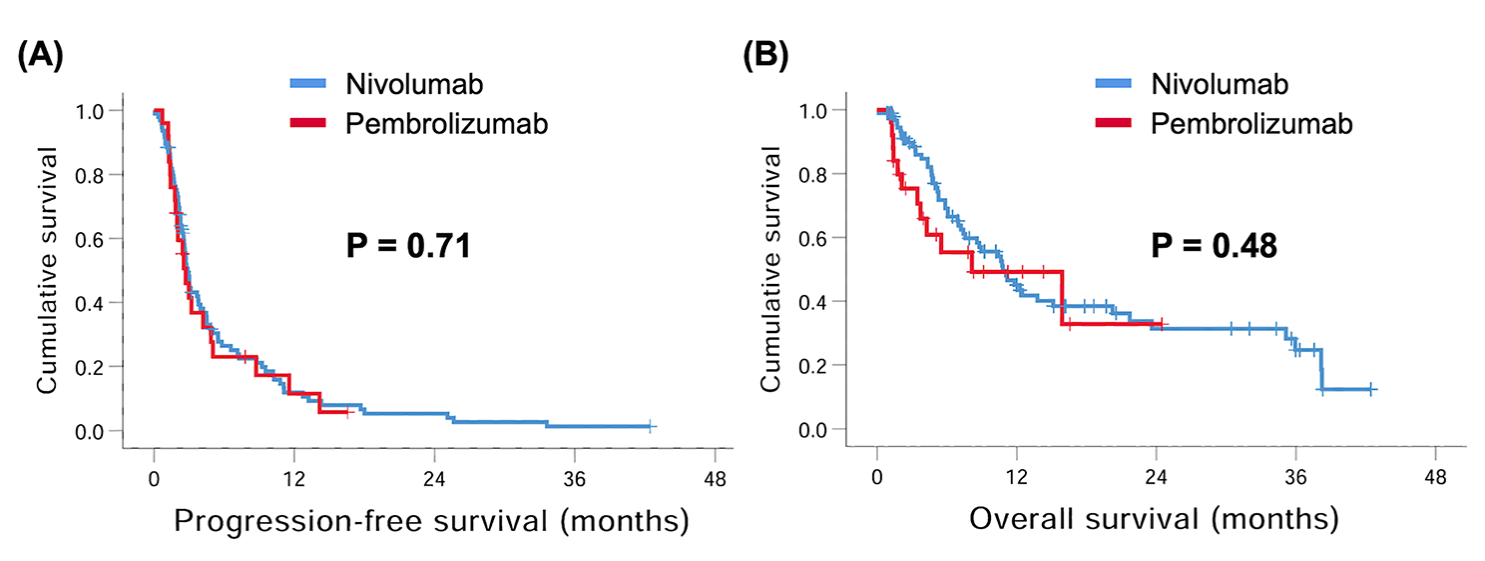
Kaplan–Meier curves for progression-free survival (PFS) and overall survival (OS) in patients with advanced hepatocellular carcinoma who received pembrolizumab or nivolumab. **(A)** PFS and **(B)** OS.

The median PFS in the pembrolizumab and nivolumab groups was 2.7 months and 2.9 months, respectively, with no significant difference (P = 0.71, Fig. 2A). The effect of pembrolizumab and nivolumab on median PFS was consistent across subgroups based on baseline characteristics except for LN metastasis, and patients with LN metastasis treated with nivolumab had better PFS than those treated with pembrolizumab (3.7 months versus 1.3 months, P = 0.001).

**Fig. 3:**
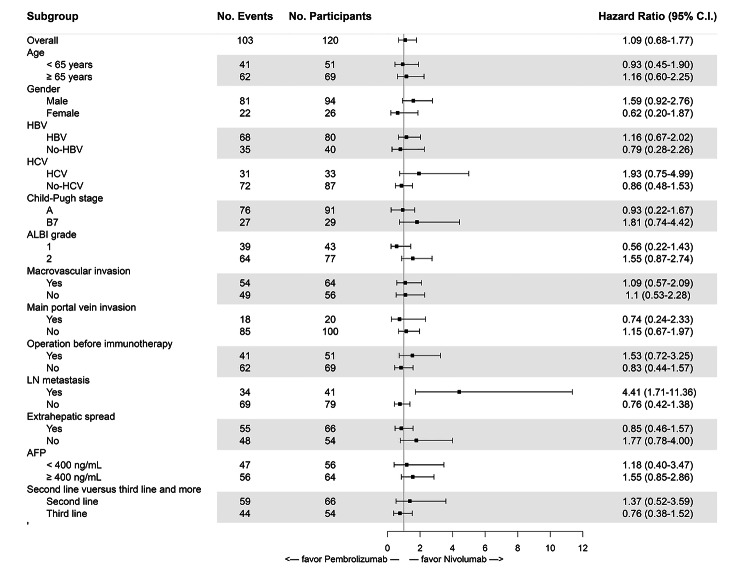
Forest plots of progression-free survival (PFS) in patient subgroups

**Fig. 4:**
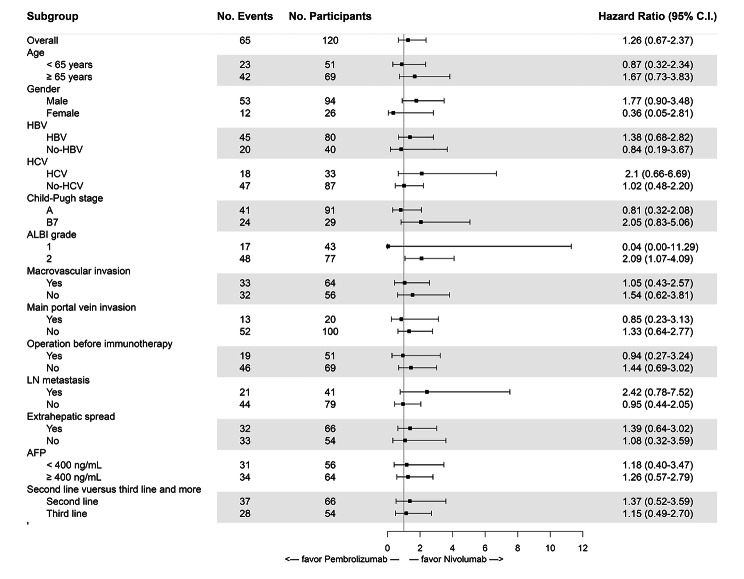
Forest plots of overall survival (OS) in patient subgroups

Although the median OS in the nivolumab group was longer than that in the pembrolizumab group (10.8 months versus 8.1 months), the difference was not statistically significant (P = 0.48, Fig. 2B). The subgroup analysis showed no difference in OS between these two groups, except for ALBI grade; for patients with ALBI grade 2, better OS was noted in the nivolumab group than in the pembrolizumab group (8.6 months versus 4.3 months, P = 0.027). The subgroup PFS and OS analyses are shown in Figs. 3 and 4, respectively.

### Safety

The most common treatment-related AEs between pembrolizumab and nivolumab groups were fatigue (12.0% versus 10.5%), rash (8.0% versus 8.4%), increased aspartate/alanine aminotransferase levels (8.0% versus 7.4%), diarrhea (4.0% versus 4.2%), pruritus (4.0% versus 4.2%), decreased appetite (4.0% versus 3.2%), hypothyroidism (4.0% versus 3.2%), decreased body weight (4.0% versus 2.1%), nausea (4.0% versus 2.1%), and hypersensitivity/infusion-related reaction (4.0% versus 2.1%). The severity of all AEs was grade 1–2, with no grade 3–4 toxicities or drug-related grade 5 AEs. Immune-modulating therapy and systemic corticosteroids were not administered to any patient for the treatment of irAEs. No patient experienced treatment interruption or dose adjustment owing to AEs in either the pembrolizumab or nivolumab group. There was no statistically significant difference in the incidence of treatment-related AEs between these two groups, and the comparison is presented in Table 3.

**Table 3:** Incidence of treatment-related adverse events in 120 patients with advanced hepatocellular carcinoma who underwent pembrolizumab or nivolumab

Adverse event	Pembrolizumab (n = 25)			Nivolumab (n = 95)		P value
	Any grades	Grade 3/4		Any grades	Grade 3/4	
Pruritis	1 (4.0%)	0 (0%)		4 (4.2%)	0 (0%)	0.96
Rash	2 (8.0%)	0 (0%)		8 (8.4%)	0 (0%)	0.95
Fatigue	3 (12.0%)	0 (0%)		10 (10.5%)	0 (0%)	0.83
Nausea	1 (4.0%)	0 (0%)		2 (2.1%)	0 (0%)	0.59
Diarrhea	1 (4.0%)	0 (0%)		4 (4.2%)	0 (0%)	0.96
Decreased body weight	1 (4.0%)	0 (0%)		2 (2.1%)	0 (0%)	0.59
Decreased appetite	1 (4.0%)	0 (0%)		3 (3.2%)	0 (0%)	0.84
Aspartate/Alanine aminotransferase increase	2 (8.0%)	0 (0%)		7 (7.4%)	0 (0%)	0.92
Hyperthyroidism	0 (0%)	0 (0%)		1 (1.1%)	0 (0%)	0.61
Hypothyroidism	1 (4.0%)	0 (0%)		3 (3.2%)	0 (0%)	0.84
Pneumonitis	0 (0%)	0 (0%)		0 (0%)	0 (0%)	1.0
Hypersensitivity/infusion-related reaction	1 (4.0%)	0 (0%)		2 (2.1%)	0 (0%)	0.59

### Patient disposition

Ten patients (40.0%) in the pembrolizumab group and 40 patients (42.1%) in the nivolumab group received subsequent therapy after pembrolizumab or nivolumab progression, including targeted therapy, chemotherapy, and ICIs (P = 0.85). Regarding targeted therapy, the most common medication in the pembrolizumab group was ramucirumab (30.0%), followed by lenvatinib (20.0%), sorafenib (20.0%), and cabozantinib (10.0%); in the nivolumab group, the most common drug was lenvatinib (27.5%), followed by regorafenib (15.0%), ramucirumab (12.5%), thalidomide (12.5%), cabozantinib (10.0%), and sorafenib (7.5%). Chemotherapy included FOLFOX (oxaliplatin, leucovorin, 5-fluorouracil), epirubicin, and gemcitabine. The most common chemotherapy regimens in the pembrolizumab and nivolumab groups were epirubicin (20.0%) and FOLFOX (30.0%), respectively. In addition, some patients continued to receive immunotherapy after progression to pembrolizumab or nivolumab, and the percentage of subsequent immunotherapy was higher in the pembrolizumab group than in the nivolumab group (50.0% vs. 17.5%), including atezolizumab plus bevacizumab, nivolumab plus ipilimumab, pembrolizumab (nivolumab group), and nivolumab (pembrolizumab group). The post-pembrolizumab and nivolumab treatment profiles are presented in Table 4.

**Table 4:** Subsequent therapy after pembrolizumab or nivolumab progression

Category	Pembrolizumab (n = 25)	Nivolumab (n = 95)	P value
Any post-nivolumab anti-cancer treatment	10 (40.0%)	40 (42.1%)	0.85
Targeted therapy			
Lenvatinib	2 (20.0%)	11 (27.5%)	
Sorafenib	2 (20.0%)	3 (7.5%)	
Regorafenib	0 (0%)	6 (15.0%)	
Ramucirumab	3 (30.0%)	5 (12.5%)	
Cabozantinib	1 (10.0%)	4 (10.0%)	
Thalidomide	0 (0%)	5 (12.5%)	
Chemotherapy			
FOLFOX	1 (10.0%)	12 (30.0%)	
Epirubicin	2 (20.0%)	7 (17.5%)	
Gemcitabine	0 (0%)	2 (5.0%)	
Immune checkpoint inhibitors			
Atezolizumab plus bevacizumab	1 (10.0%)	4 (10.0%)	
Nivolumab plus ipilimumab	2 (20.0%)	1 (2.5%)	
Pembrolizumab	0 (0%)	2 (5.0%)	
Nivolumab	2 (20.0%)	0 (0%)	

## Discussion

Our study revealed a real-world comparison of the efficacy and safety of pembrolizumab and nivolumab for the management of advanced HCC. The ORR was approximately 8% in the pembrolizumab and nivolumab groups, and the profile of treatment response was similar, including SD, PD, and DCR. There was no statistically significant difference in PFS and OS, although the nivolumab group had a longer OS than the pembrolizumab group (10.8 months versus 8.1 months). In addition, the safety profile demonstrated good tolerability without any grade 3–4 toxicities, treatment interruptions, or dose adjustments. The percentage of subsequent therapy after progression to pembrolizumab or nivolumab was equal between these two groups, contributing to the lack of statistical difference in OS. Overall, our study showed that the efficacy and safety of pembrolizumab and nivolumab are comparable in the management of patients with pretreated HCC in real-world practice.

Pembrolizumab and nivolumab are both monoclonal antibodies that target the PD-1 receptor on T cells and have been approved for the treatment of advanced HCC. Based on the results of clinical trials, pembrolizumab has demonstrated consistent efficacy compared with nivolumab in patients with HCC. In the Phase III KEYNOTE-394 trial, pembrolizumab was found to improve OS compared to placebo in patients with advanced HCC who had previously been treated with sorafenib or chemotherapy [13]. The median OS was 14.6 months in the pembrolizumab group compared to 13.0 months in the placebo group. The ORR in the pembrolizumab group was 12.7% with a DCR of 51%. In contrast, in the Phase II CheckMate-040 trial (Asian cohort analysis), nivolumab demonstrated an ORR of 14%, disease control rate of 55%, and median OS of 15.1 months in patients with advanced HCC who had previously been treated with sorafenib [10]. In terms of safety, both drugs have similar adverse effects such as fatigue, diarrhea, and rash.

Growing real-world evidence has confirmed the efficacy and safety of nivolumab or pembrolizumab alone for the treatment of advanced HCC [20–23]. However, comparisons between pembrolizumab and nivolumab for the management of pretreated HCC are limited. Kuo et al. reported a retrospective study of 115 patients who received nivolumab or pembrolizumab treatment and showed that the pembrolizumab group had a higher ORR than the nivolumab group (38.1% vs. 15.1%, respectively) [24]. In addition, pembrolizumab performed a superior OS than nivolumab (34.9 months versus 9.5 months), but the incidence of AEs was comparable in both groups. These findings are slightly different from those of our study and may be attributed to several reasons. First, combination with MKIs was allowed in the former study, including 53.4% in the nivolumab group and 71.4% in the pembrolizumab group, which may have resulted in a better ORR and OS in the pembrolizumab group than in the nivolumab group. In contrast, only pembrolizumab or nivolumab was enrolled in our study, contributing to the lack of statistically significant differences in ORR, PFS, and OS between the two groups. Second, nearly 30% and 60% of pembrolizumab or nivolumab were used as first- and second-line treatments in the former study, respectively; this may have caused the higher ORR and longer OS compared to the results of the KEYNOTE-224 and KEYNOTE-394 trials. However, in our study, pembrolizumab or nivolumab was prescribed as second-line (55%) or third-line and later therapy (45%), which was the real subsequent therapy after progression of the MKIs.

Microsatellite instability high (MSI-H) or mismatch repair deficient (dMMR) has been regarded as potential biomarkers to predict treatment response and survival benefit to ICIs. Based on the results of KEYNOTE-177 and KEYNOTE-158, pembrolizumab provided clinically meaningful antitumor activity, including high ORR, long duration of response, and manageable safety in patients with advanced colorectal cancer and non-colorectal cancers [25–27]. Therefore, pembrolizumab has been approved for the treatment of advanced MSI-H solid tumors. In HCC, the frequency of MSI-H or dMMR was rare, around 0–3% according to previous studies [28–30]. However, patients with liver cirrhosis had higher rate of MSI than those without liver cirrhosis [31]. Furthermore, animal model also demonstrated that hepatocyte-specific disruption of MutS homologue 2 (MSH2) contributed to the development of HCC [32]. In conclusion, although the incidence of MSI-H or dMMR was rare in HCC, inflammation-mediated dysfunction of the MMR pathway may result in increased mutations during hepatitis-associated carcinogenesis, and these findings also explained the reason about the benefit of ICIs in the treatment of HCC. In our cohort, only one patient was mentioned to have dMMR and he received pembrolizumab with a response of PR.

In our study, some patients received ICIs as a subsequent therapy, even after progression to pembrolizumab or nivolumab. In general, patients may receive other ICIs based on different mechanisms, such as programmed cell death-1 **(**PD-1**)** versus programmed death-ligand 1 (PD-L1) or based on the treatment course (nivolumab for the pembrolizumab group or pembrolizumab for the nivolumab group). Increasing evidence has demonstrated a re-challenge of immunotherapy in the management of HCC [33, 34]. Schenier et al. demonstrated that the ORR was approximately 22%, with a median time to progression of 5.2 months, indicating that ICI rechallenge was relatively safe and contributed to a treatment benefit in a meaningful proportion of patients with HCC.

In addition to ICI rechallenge, targeted therapy and chemotherapy were also used after progression to nivolumab or pembrolizumab. Lenvaitnib was one of the most common used targeted therapy and our previous study showed similar PFS and OS in patients with lenvatinib as second line, third line and later line [35]. Ramucirumab has been approved for the treatment of HCC in patients with AFP ≥ 400 ng/mL based on the results of REACH-2 trial [8]. An expansion cohort of REACH-2 trial demonstrated the efficacy of ramucirumab following non-sorafenib systemic therapies [36]. The median PFS and OS were 1.7 months and 8.7 months, respectively; but the ORR was up to 25% in patients who underwent ICIs (4 patients with PR/16 patients), indicating the experience of prior ICIs did not affect the efficacy of ramucirumab in the management of HCC. Although chemotherapy is not suggested as first-line systemic therapy, it is still one of the most important treatment modalities for advanced HCC. The mechanism of chemotherapy is different from targeted therapy and immunotherapy, and many chemotherapy regimens were mentioned to provide survival benefit in previous studies, such as FOLFOX, GEMOX (gemcitabine plus oxaliplatin), and XELOX (oxaliplatin plus capecitabine) [37–39]. In our cohort, among patients with anti-cancer treatment after progression to nivolumab or pembrolizumab, near 50–60% of patients received targeted therapy and near 30–40% of patients underwent chemotherapy.

Our study has several limitations. First, it was retrospectively designed with a relatively small sample size, which may have resulted in a selection bias. Second, the patient numbers in the pembrolizumab and nivolumab groups were not balanced, contributing to a low statistical power. Third, the follow-up period may not have been long enough, making it difficult to conclude a statistical difference in some potential clinicopathological parameters. Fourth, many targeted therapies or chemotherapy regimens are not reimbursed by the national health insurance system in Taiwan, which might limit the use of subsequent therapies after progression to pembrolizumab or nivolumab, contributing to the difference in OS. However, to our knowledge, this is one of the few studies to explore the real-world efficacy and safety of pembrolizumab versus nivolumab in the management of pretreated HCC in clinical practice. To better understand the efficacy and toxicity of pembrolizumab and nivolumab, an international multi-center propensity score-matched retrospective cohort study may be feasible and helpful in reducing selection bias.

## Conclusions

The results of our study show that the efficacy and safety of pembrolizumab and nivolumab are comparable in the management of patients with pretreated HCC in real-world practice. More high-quality, well-designed prospective studies with larger sample sizes are needed to validate our findings.

## Data Availability

The datasets used and analyzed in the current study are available from the corresponding author upon reasonable request.

## Abbreviations

HCC: hepatocellular carcinoma
MKI: multi-kinase inhibitor
ICI: immune checkpoint inhibitor
OS: overall survival
ORR: objective response rate
PFS: progression-free survival
AFP: alpha-fetoprotein
DCR: disease control rate
AASLD: American Association for the Study of Liver Disease
BCLC: Barcelona Clinic Liver Cancer
ALBI: albumin-bilirubin
MRI: magnetic resonance imaging
CT: computed tomography
RECIST: Response Evaluation Criteria in Solid Tumor
Q2W: every 2 weeks
Q3W: every 3 weeks
AE: adverse event
irAE: immune-related adverse event
CTCAE: Common Terminology Criteria for Adverse Event
HR: hazard ratio
CI: confidence interval
PR: partial response
SD: stable disease
PD: progressive disease
PD-1: programmed cell death-1
PD-L1: programmed death-ligand 1
MSI-H: microsatellite instability high
dMMR: mismatch repair deficient
MSH2: MutS homologue 2
